# Growth Hormone Deficiency Following Traumatic Brain Injury in Pediatric and Adolescent Patients: Presentation, Treatment, and Challenges of Transitioning from Pediatric to Adult Services

**DOI:** 10.1089/neu.2022.0384

**Published:** 2023-06-27

**Authors:** Tamara L. Wexler, Kent Reifschneider, Philippe Backeljauw, Javier F. Cárdenas, Andrew R. Hoffman, Bradley S. Miller, Kevin C. J. Yuen

**Affiliations:** ^1^ Department of Rehabilitation Medicine, NYU Langone Health, New York, New York, USA.; ^2^ Division of Endocrinology, Diabetes, and Metabolism, Perelman School of Medicine, University of Pennsylvania, Philadelphia, Pennsylvania, USA.; ^3^ Children's Hospital of The King's Daughters, Eastern Virginia Medical Center, Norfolk, Virginia, USA.; ^4^ Cincinnati Children's Hospital Medical Center, University of Cincinnati College of Medicine, Cincinnati, Ohio, USA.; ^5^ Barrow Concussion and Brain Injury Center, Barrow Neurological Institute, University of Arizona College of Medicine and Creighton School of Medicine, Phoenix, Arizona, USA.; ^6^ Department of Medicine, Division of Endocrinology, Metabolism and Gerontology, Stanford University School of Medicine, Stanford, California, USA.; ^7^ Department of Pediatrics, Division of Pediatric Endocrinology, University of Minnesota Medical School, M Health Fairview Masonic Children's Hospital, Minneapolis, Minnesota, USA.; ^8^ Barrow Pituitary Center, Barrow Neurological Institute, University of Arizona College of Medicine and Creighton School of Medicine, Phoenix, Arizona, USA.

**Keywords:** growth hormone deficiency, post-traumatic hypopituitarism, transition of care, traumatic brain injury

## Abstract

Traumatic brain injury (TBI) is increasingly recognized, with an incidence of approximately 110 per 100,000 in pediatric populations and 618 per 100,000 in adolescent and adult populations. TBI often leads to cognitive, behavioral, and physical consequences, including endocrinopathies. Deficiencies in anterior pituitary hormones (e.g., adrenocorticotropic hormone, thyroid-stimulating hormone, gonadotropins, and growth hormone [GH]) can negatively impact health outcomes and quality of life post-TBI. This review focuses on GH deficiency (GHD), the most common post-TBI pituitary hormone deficiency. GHD is associated with abnormal body composition, lipid metabolism, bone mineral density, executive brain functions, behavior, and height outcomes in pediatric, adolescent, and transition-age patients. Despite its relatively frequent occurrence, post-TBI GHD has not been well studied in these patients; hence, diagnostic and treatment recommendations are limited. Here, we examine the occurrence and diagnosis of TBI, retrospectively analyze post-TBI hypopituitarism and GHD prevalence rates in pediatric and adolescent patients, and discuss appropriate GHD testing strategies and GH dosage recommendations for these patients. We place particular emphasis on the ways in which testing and dosage recommendations may change during the transition phase. We conclude with a review of the challenges faced by transition-age patients and how these may be addressed to improve access to adequate healthcare. Little information is currently available to help guide patients with TBI and GHD through the transition phase and there is a risk of interrupted care; therefore, a strength of this review is its emphasis on this critical period in a patient's healthcare journey.

## Introduction

Traumatic brain injury (TBI) is an increasingly common occurrence in individuals of all ages, and is one of the main causes of disability, morbidity, and mortality.^1,2^ Following single or multiple TBIs, transient or permanent hypopituitarism may develop in the acute and/or the chronic phase. Growth hormone deficiency (GHD) is often reported as the most common pituitary hormone deficiency post-TBI, followed by hypogonadism, thyroid-stimulating hormone (TSH) deficiency, adrenocorticotropic hormone (ACTH) deficiency, and diabetes insipidus (DI).^3-5^ The pathophysiology of post-traumatic hypopituitarism (PTHP) may be related to the primary injury, including direct trauma to the hypothalamus or pituitary gland and compressive effects from surrounding structures. Secondary effects, which may also contribute to PTHP, are mainly related to the interplay of a complex and ongoing cascade of specific molecular/biochemical events, including those related to inflammation, ischemia, and hypoxia.^6-8^ The aim of this review is to discuss TBI and post-TBI GHD in children, adolescents, and individuals in the transition phase to adulthood, with an emphasis on ways in which diagnosis, management, and treatment recommendations can change during the transition from pediatric to adult healthcare systems.

## Methods

In this narrative review, authors performed a comprehensive literature search in PubMed and as a group, arrived at a cohesive description and set of recommendations for diagnosing and treating post-TBI GHD. As part of this review, authors investigated reported prevalence rates of post-TBI PTHP and GHD. Search terms used were “TBI” or “traumatic brain injury” and “GHD” or “growth hormone deficiency” or “PTHP” or “post traumatic hypopituitarism.” (This review was not intended as a meta-analysis, as there is a lack of sufficient literature to power such an analysis.)

Studies included in the analysis of PTHP prevalence rates were those that reported only anterior pituitary deficiencies in pediatric and adolescent patients in the chronic phase after TBI. Studies that reported non-anterior pituitary deficiencies, including diabetes insipidus, were excluded. If possible, for studies that reported both anterior and non-anterior pituitary deficiencies, prevalence rates for anterior pituitary deficiencies were extracted and reported here. GHD was required to be diagnosed using a stimulation test, adhering to the cutoff value of <10 μg/L suggested in the Growth Hormone (GH) Research Society guidelines published in 2000.^9^ For studies that reported GHD prevalence rates based on more or less stringent thresholds, these guidelines were retroactively applied and the resulting prevalence rates are reported.

Studies included in the analysis of GHD prevalence rates were those that reported rates of GHD in pediatric and adolescent patients in the chronic phase post-TBI. As above, reports of GHD were limited to those that were diagnosed using a stimulation test and that adhered to the cutoff value of <10 μg/L suggested in the GH Research Society guidelines published in 2000.^9^ For studies that reported GHD prevalence rates based on more or less stringent thresholds, these guidelines were retroactively applied and the resulting prevalence rates are reported.

The results of these analyses are presented in Supplementary Table S1 and Supplementary Table S2. Although there was insufficient literature to power a meta-analysis, these analyses are nonetheless beneficial for the understanding of PTHP and GHD following TBI in pediatric and adolescent patient populations.

### TBI events in children and adolescents

In recent years, TBI has been increasingly recognized as a major cause of morbidity and mortality in children and adolescents.^10,11^ A TBI is defined as a change in brain function or pathology as a result of an external force.^12^ It is accompanied by impairments in neurologic function (e.g., alterations in balance, weakness, altered vision) or mental disturbances (e.g., confusion, disorientation).^13^ TBI may also be accompanied by a number of additional sequelae, including loss of consciousness, neuroinflammation, epilepsy/seizures, hydrocephalus, brain swelling, headache, olfactory dysfunction, motor disturbances, cognitive deficits, attention and memory difficulties, sleep problems, and endocrine changes.^6,8,13–23^

One meta-analysis estimated that the occurrence of TBI is approximately 110 per 100,000 in pediatric populations (< 15 years of age) and 618 per 100,000 in adolescent and adult populations.^24^ In older adults and young children, TBI is most frequently caused by falls, while in young adults, TBI is most frequently caused by motor vehicle accidents.^25^ Other increasingly reported causes of TBI in young adults include domestic abuse or violence and sports injuries.^25,26^ The rate of TBI may be up to twice as common in males, although it is difficult to estimate the precise incidence in any given population due to underreporting of mild and moderate injuries.^24,27^

Altered brain function can stem from head injuries of any severity, ranging from concussion and mild TBI to severe TBI. Concussion is largely a clinical diagnosis, relying on balance testing and neuropsychological evaluations to indicate cognitive deficits.^12^ The severity of TBI is typically classified using the Glasgow Coma Score (GCS), which assesses performance in three domains: eye, motor, and verbal. Individual scores from each domain are added together for an overall GCS score.^13^ Scores between 3 and 8 indicate severe TBI, while scores of 9 to 12 and 13 to 15 indicate moderate and mild TBI, respectively (Table 1).^25^ While the GCS system is useful for the initial evaluation and stratification of patients with TBI, the system is limited by its high inter-rater score variability, can be confounded by sedation or intubation in a hospital setting, may be unreliable in patients between 9 and 12 years of age, and does not accurately reflect pathological or anatomical differences in TBI.^13,28,29^ Scores are especially variable in young children, at least in part due to difficulties assessing the verbal score component^29^; therefore, a pediatric GCS assessment incorporating age-appropriate modifications has also been developed.^28,30^ Some studies suggest that the GCS motor subscore may perform similarly to, if not better than, the full GCS when predicting severe head trauma in adults; however, this requires additional investigation and validation in pediatric populations.^28,31,32^

**Table 1.** Measurements of TBI Severity

Measurement system	Mild TBI	Moderate TBI	Severe TBI
GCS	13-15	9-12	3-8
Amnesia	≤ 24 h	> 24 h to <7 days	≥ 7 days
Loss of consciousness	0-30 min	> 30 min to <24 h	≥ 24 h

TBI, traumatic brain injury; GCS, Glasgow Coma Score.

An alternative to the GCS is the Full Outline of UnResponsiveness (FOUR) score, which includes four components: eye, motor, brain stem, and respiratory scores. The advantages of the FOUR score over the GCS system are that it has been adapted for the pediatric population and has been shown to have good inter-reporter variability when evaluating pediatric trauma severity.^33^ The FOUR score may hold an advantage over the GCS in subcategorizing patients with low GCS scores and in evaluating patients in intensive care unit (ICU) settings, although these observations are based on limited clinical data; therefore, additional studies are needed.^34^ Other informative clinical measures of severity include duration of post-traumatic amnesia and witnessed loss of consciousness (Table 1).^29,35,36^ Of note, the endocrinopathies addressed here can occur after any degree of concussion/TBI.

The time after a TBI event is typically stratified into the acute and chronic phases. The acute phase is defined as the 2 to 4 weeks following the injury.^25,27,37^ Acute TBI may be associated with symptoms including irritability, headache, dizziness, loss of consciousness, olfactory dysfunction, amnesia, confusion, and seizures.^13,16,17,21,23^ Adrenal insufficiency may occur in the acute phase and has been reported in 2 to 43% of children with TBI.^27^ Adrenal disturbances may be characterized by hypoglycemia, fatigue, confusion, hypotension, and hyponatremia.^11,25^ Deficiencies in the adrenal axis should be addressed and treated immediately during the acute phase, as these deficiencies can be life-threatening and may also correlate with neurological dysfunction.^11,25,27,38^ DI, which, when it occurs, almost always presents in the acute phase, is uncommon in children who have experienced TBI.^10^ When DI does occur, it is rarely permanent but should be addressed immediately.^7,8^ Evaluation of TSH and free T4 for identification of TSH deficiency during the acute phase can result in unreliable readings, as thyroid hormone concentrations can change during stress, illness, and hospitalization.^19,39,40^ Therefore, it is not recommended that thyroid function be evaluated until at least 3 months post-TBI.^41^

Deficiencies in other anterior pituitary hormones, including gonadotropins and GH, may present during the acute phase; however, the relevance of these deficiencies in the acute phase is unclear and testing is often deferred until at least 3 to 6 months post-TBI.^25,27^ The presence or lack of pituitary deficiencies in the acute phase does not reliably predict the development of hypopituitarism in the chronic phase; even testing for acute adrenal insufficiency, the one recommended pituitary test in the acute period, does not predict chronic adrenal insufficiency in either adults^42-44^ or children.^45^ This is because deficits identified within the first year following the TBI event may be transient and recover over time.^25^ As the focus of this article is GHD in the chronic phase post-TBI, the acute phase will not be discussed further. Diagnosis and treatment of additional pituitary hormone deficiencies in the chronic phase will be discussed in later sections.

There is no clear threshold delineating the end of the acute phase and the beginning of the chronic phase. However, it is suggested that by 3 months post-TBI, a patient has entered the chronic phase, during which a wide range of physical, behavioral, and cognitive outcomes can occur.^25,27^ Physical symptoms associated with TBI in the chronic phase include fatigue, headache, dizziness, nausea, and sleep pattern alterations.^11,13,21,23^ Less commonly, TBI may be associated with alterations in or loss of vision, hearing, or smell^21^; olfactory dysfunction may be correlated with the severity of TBI.^15^ Patients who have experienced a TBI event may be at increased risk of depression, anxiety, and post-traumatic stress disorder (PTSD) and may experience personality changes, including increased irritability, impulsivity, and apathy.^13,19,21,46^ Importantly, mental and physical symptoms of depression, TBI, and PTSD may overlap, complicating the diagnosis.^36,46^ Patients may also experience word-finding difficulties, attention and memory disturbances, social isolation, increased instances of being bullied, and reduced academic performance.^13,17,47-49^

There is considerable debate around the effect of age on TBI outcomes. For example, children display increased levels of neuronal plasticity, which could aid in a more rapid and robust recovery following TBI.^7,45^ Conversely, TBIs that occur during specific times in a child's life, particularly during critical times of cognitive and neuronal development and during physical and sexual maturation, may therefore have a greater impact on post-TBI outcomes.^17,50^

### Post-TBI hypopituitarism in children and adolescents

A subset of patients with TBI will experience PTHP, defined as a deficiency in one or more pituitary hormones following a traumatic event to the brain.^37^ Severe TBI seems to correlate with the highest risk of PTHP^11,37,51^; however, PTHP can occur with any severity of TBI or even with repetitive concussions.^52–56^

Several mechanisms play a role in the pathophysiology of PTHP. Shearing and compression forces, which occur during the TBI event, can lead to pituitary tissue necrosis, reducing pituitary hormone production and secretion.^27^ Stalk torsion or displacement caused by TBI can lead to disruption of blood flow to the pituitary gland.^11,27^ TBI-induced edema, hemorrhage, or increased intracranial pressure can cause pituitary compression and dysfunction.^11,25^ Patients with TBI also display increased glutamate excitotoxicity, which can lead to neurocognitive defects, particularly during key developmental periods.^6,25,57^ Finally, TBI can induce inflammation, ischemia, oxidative stress, and cytotoxicity in the nervous system. These factors may accelerate necrosis of neuronal cells and can increase blood–brain barrier permeability, resulting in increased pituitary antigen exposure and the potential development of autoantibodies and hypophysitis.^6,14,25,27,37,58^

As part of this review, a retrospective analysis was performed on studies that reported PTHP prevalence rates in children and adolescents post-TBI. This analysis revealed that PTHP may be present in 10 to 58% of pediatric and adolescent patients at any time during the chronic phase of TBI (Supplementary Table S1).^26,45,59–66^ Studies selected for inclusion in this analysis were those that reported on anterior pituitary deficiencies during the chronic phase. Reports of GHD were restricted to those diagnosed using a stimulation test and a threshold value GH <10 μg/L.^9^ When studies reported on both anterior and non-anterior pituitary deficiencies, prevalence rates for pituitary deficiencies of interest were extracted from a larger data set if possible (detailed in Supplementary Table S1). The large range of PTHP prevalance rates reported here is likely indicative of differences in study design, variability and low reproducibility of testing methods, timing of pituitary axis measurements, and injury severity.^7^

Age and sex of study participants may also contribute to variations in reported PTHP prevalence rates. One study suggested that the highest rates of pituitary hormone deficiencies in children occur between 7 and 11 years of age.^10^ While overall rates of PTHP seem to be similar in males and females,^19^ sex-specific diagnoses may vary based on the specific hormone being investigated. For example, males appear to present with increased rates of GHD relative to females.^46^ Larger studies are needed to confirm these findings; however, this discrepancy may reflect the increased referral, evaluation, and treatment of boys for growth deceleration and short stature compared with girls.^46,67^ While reports of endocrine dysfunction in adults after TBI typically favor isolated pituitary abnormalities, reports in children favor multiple pituitary deficiencies. This may reflect selection bias (multiple deficiences often correlate with a more severe presentation and increased reporting) and varying diagnostic and referral practices between adult and pediatric populations.^64^ In general, adults and children tend to present with comparable overall rates of pituitary deficiencies, and in both populations, GH is the most common pituitary hormone affected.^22^

Based on evaluation of existing reports, between 9.1% and 47.8% of children and adolescents with TBI may develop GHD (Supplementary Table S2).^26,45,60-63,65-67^ As in Supplementary Table S1, reports of GHD in Supplementary Table S2 were restricted to those diagnosed using a stimulation test and a threshold value GH <10 μg/L.^9^ For studies that reported GHD prevalence rates based on different criteria, these guidelines were retroactively applied, where possible (for more details, see Supplementary Table S2). Similar to the reports on PTHP already noted, this broad range is likely due to differences in the timing and methodology of GHD testing. Clinical consequences of GHD may include reduced adult height, decreased lean body mass and muscle strength, reduced metabolic rate, increased abdominal fat, decreased quality of life (QOL), cognitive issues, and memory and attention deficits.^25,68–71^ There is evidence to suggest that TBI with GHD confers worse risk for cognitive issues (e.g., poorer verbal learning, verbal short-term memory, and attention) than does TBI without GHD.^11,25,37^ Additional anterior pituitary abnormalities observed in children and adolescents in the chronic phase post-TBI may include ACTH deficiency (reported at 2-43%), gonadotropin deficiency (reported at 6-16%), TSH deficiency (reported at 2-33%), and precocious puberty (reported at 2-16%).^7,11,27^

### Testing for GHD in pediatric and adolescent patients with TBI

Consensus guidelines published in 2005 recommend that adults with moderate to severe TBI undergo endocrine screening.^72^ However, there are currently no screening guidelines specific to children or adolescents for investigating endocrine dysfunction after a TBI.^10^

In adults, GHD is diagnosed based on biochemical assessment of the GH axis using well-defined thresholds. In children, GHD can be strongly indicated based on insulin-like growth factor I (IGF-I) concentrations, height velocity, or other auxologic features, but is often confirmed with biochemical testing.^8,65,73,74^ Changes in growth are not universally observed in these patients.^7,63^ Given that GHD can be transient and that secretory patterns may change over time, children should undergo routine GH testing (e.g., every 6 months) for at least 1 year post-TBI.^7,11,27^ While some GH stimulation tests have been applied to younger populations (detailed below), sensitive and specific cut points for the biochemical detection of GHD in children are currently debated, and results of these tests should be interpreted with caution.^75^ GH stimulation tests should be performed only after treatments for all other pituitary hormone deficiencies have been established, as changes in other pituitary hormones can affect GH axis testing results.^76,77^

The insulin tolerance, growth hormone releasing hormone (GHRH) + arginine, GHRH + growth hormone-releasing peptide 6 (GHRP-6), glucagon, arginine, clonidine, levodopa (l-DOPA), and macimorelin tests have all been used to detect GHD in pediatric and adolescent patients.^7,60,65,67,74,78,79^ Stimulation test thresholds ranging from <5 to <10 μg/L have been suggested as indicative of GHD in children,^73,80,81^ with the current consensus value listed as <10 μg/L.^9^

The use of several of these tests is limited by safety concerns and availability. The insulin tolerance test (ITT) is used less frequently in the U.S. due to its potential to cause complications of hypoglycemia (especially in patients with undiagnosed adrenal insufficiency) and contraindication in patients with a history of cardiac disease or seizures.^23,25,37,76,82^ The GHRH + arginine and GHRH + GHRP-6 tests cannot detect GHD of hypothalamic origin and are unavailable in many countries.^25,74^ In the U.S., recombinant GHRH was removed from the market in July 2008^11,76^ and GHRP-6 was never available. It is important to note that the diagnostic accuracy of stimulation tests may be reduced in patients with glucose intolerance or obesity; body mass index (BMI)-adjusted thresholds have been suggested for some tests, such as the GHRH + arginine test.^25,76,83,84^ Given the availability, safety, and contraindications for the above-mentioned stimulation tests, the glucagon stimulation test (GST) is now emerging as the preferred GHD diagnostic test for adults.^11^ The preferred diagnostic test in children is less clear.

Macimorelin is an orally administered GH secretagogue receptor agonist that binds the ghrelin receptor, stimulating dose-dependent GH production. Macimorelin was approved by the U.S. Food and Drug Administration (FDA) for diagnosis of adult GHD in December 2017. The original approval listed a cutoff value of 2.8 μg/L, but a higher cutoff value of 5.1 μg/L was subsequently demonstrated to increase GHD detection sensitivity.^76,85^ Thus far, there is no indication that different cutoff values will be required for patients with different BMIs.^76^ Advantages of the macimorelin test include easy oral dosing and fewer blood draws^74^; however, macimorelin is a more expensive diagnostic agent than other options.^76^ Macimorelin is currently being investigated for the diagnosis of GHD in pediatric populations, with initial reports revealing that the compound is both safe and well tolerated.^78^ Other agents, including LUM-201, are also being evaluated for the diagnosis of GHD in children.^86^

As IGF-I concentrations are partially GH-dependent, serum IGF-I measurements have been used to diagnose GHD. Very low IGF-I concentrations are suggestive of GHD and it has been reported that IGF-I concentrations >0 standard deviation score (SDS) make GHD unlikely at any age.^74,87^ However, IGF-I concentrations are also influenced by a variety of other factors and should be used as a diagnostic tool with caution, as there is a large overlap in IGF-I production between patients with GHD and those who are GH sufficient, particularly in very young children.^3,74,88,89^ IGF-I concentrations may be normal in patients who fail provocative GH testing; therefore, serum IGF-I is not always a reliable indicator of GHD.^18,45,74,89-91^

While similar stimulation testing options exist for patients in the pediatric, adolescent, and adult populations, GH secretion varies considerably during childhood and adolescence. Thus, while diagnostic thresholds have been well defined for adult GHD, appropriate thresholds for the diagnosis of GHD in childhood and adolescence are currently debated.^92^ Previous consensus guidelines suggest that a peak stimulated GH concentration <10 μg/L indicates the presence of GHD in children. However, this threshold value does not take into account sex, age, BMI, or the specific stimulation test used.^9,81,92^ Sex steroids induce GH secretion, and children in puberty are less likely to test as GH-deficient compared with prepubertal children.^74,93^ While the effectiveness of sex steroid priming is still debated, priming may improve the specificity of some GH stimulation tests in prepubertal children^74^; guidelines published in 2016 suggest the use of priming (with oral β-estradiol or intramuscular testosterone) in prepubertal boys >11 years of age and prepubertal girls >10 years of age with an expected adult height within -2 standard deviation (SD) of the normative reference population.^81^

### Treatment of GHD in pediatric and adolescent patients

Patients with PTHP must have other (non-GH) pituitary hormone deficiencies diagnosed and treated before assessment of possible post-TBI GHD.^76^ In addition to decreased height velocity, adolescents and young adults with severe GHD report decreased QOL related to physical health and emotions. One study in 16- to 25-year-old participants reported that these outcomes can be detected using a generic QOL questionnaire but not using the disease-specific QoL-AGHDA (Quality of Life-Assessment of Growth Hormone Deficiency in Adults) questionnaire, indicating that the disease-specific questionnaire may not be applicable in adolescents and young adults with GHD.^94^

If the presence of GHD is biochemically confirmed, GH treatment in children and adolescents can improve height outcomes, depression, memory, information processing speed, executive function, and QOL.^68,95–100^ Treatment with GH can also improve bone mineral density, decrease fat mass, increase lean muscle mass, and improve cardiopulmonary functional capacity.^69,71,101^ However, GH replacement may also unmask underlying cortisol or thyroid hormone insufficiencies, necessitating close monitoring of the adequacy of glucocorticoid and thyroid hormone replacement following initiation of GH therapy.^9,77,81^

Dosing of GH in pediatric patients is currently recommended at 22 to 35 μg/kg/day, with subsequent titration to normalize IGF-I concentrations to between 0 and +2 SDS.^76,81^ Titration should also take into account possible side effects, which can include headaches, arthralgia, edema, and insulin resistance.^76,102^

It is important to consider the timing of GH replacement in children when it is used in conjunction with sex hormone replacement, as this can affect the attainment of adult height. During puberty, increases in circulating GH, IGF-I, and sex hormones lead to accelerated height velocity but also to estrogen-induced epiphyseal fusion.^93,103,104^ To ensure that adult height is obtained, GH treatment should start as early as possible after the diagnosis of GHD.^105^ Some studies have suggested that adult height may also be achieved by increasing the GH dose during puberty (to mimic the pubertal increase in GH secretion observed in individuals who are GH sufficient) or by delaying puberty using gonadotropin-releasing hormone analogs.^103,104,106^ It is important to note that oral estrogen-based contraceptives may reduce the effects of GH replacement therapy in women.^77,81,107^

Long-acting GH (LAGH) options have been developed with the aim of improving patient experiences, adherence, and outcomes. These GH products are modified to allow for rapid absorption into and/or delayed removal from the circulation.^73,76,108,109^ In the U.S., only one such product is currently approved for use in pediatric patients with GHD: once-weekly lonapegsomatropin-tcgd, a transiently PEGylated LAGH option, was approved by the FDA in August 2021 at a recommended dose of 0.24 mg/kg body weight for pediatric patients age ≥1 year who weigh at least 11.5 kg.^110^ Additional LAGH options have been approved outside of the U.S. for the treatment of pediatric GHD, including somatrogon (Canada, Australia, Japan, and the European Union), somatropin (South Korea), and PEGylated somatropin (China).^108,109,111^ Finally, somapacitan, a reversible albumin-binding GH analog has been approved for adult GHD in the U.S. (August 2020) and Europe (March 2021) and is currently being studied in pediatric populations.^109^

Patients who have experienced a TBI event should also receive cognitive behavioral therapy and/or counseling to address the effects of anxiety, depression, memory issues, and sleep issues.^20,112,113^ Speech therapy (for cognitive deficits, including for executive function issues), physical therapy (for vestibular deficits), and exercises to help rehabilitate vision and balance may also be beneficial.^20,114^

Ideally, a multi-disciplinary team of providers will be involved in the management and treatment of patients with TBI, particularly those with GHD (Table 2). Patients who experience a TBI may first present to a primary care physician (PCP) or trauma ICU team, depending on the severity of the injury. PCPs and other physicians caring for patients with TBIs should refer patients to a neurologist to address issues in mental status, memory, cognition, and reflexes; a physical medicine and rehabilitation (PM&R) specialist for diagnosis and treatment of speech, balance, gait, and other post-TBI problems; and a behavioral health specialist to manage behavioral problems and changes.^20,115^ Providers should also refer individuals with moderate to severe TBI and those with chronic, persistent symptoms to an endocrinologist with experience evaluating and treating pituitary deficiencies such as GHD to address potential TBI-related hormonal deficiencies.^11^ Endocrinologists should be a part of the care team for any patients with GHD or other pituitary deficiencies.^72^ The authors also recommend that endocrinologists and other providers seeing patients with hormonal deficiencies obtain a thorough medical history, including questions about TBI history, in order to appropriately evaluate pituitary deficiencies. Having a core multi-disciplinary care team and clear referral pathways is imperative in facilitating the screening of possible endocrinopathies and all phases of the recovery process in patients with TBI and GHD. It may also be beneficial to have one provider serve as the point of contact and care coordinator for patients.

**Table 2.** Specialties and Subspecialties That May Be Involved in the Short-Term and/or Long-Term Care of Patients With TBI and GHD

Initial presentation	Additional referral categories	Examples of providers
ICU team PCP	Endocrinology	Endocrinologist
	Neurology	Neurologist Neurosurgeon
	PM&R	PM&R specialist Occupational therapist Speech therapist Physical therapist
	Behavioral health	Behavioral health specialist Cognitive rehabilitation specialist Psychotherapist

Patients may present to either a PCP or an ICU team, or later to another specialty, depending on the severity of the TBI. PCPs and other providers caring for patients with TBIs should refer patients to several additional specialties depending on injury severity and symptoms: endocrinology (to assess pituitary hormone deficiencies; for most hormonal deficiencies, these referrals should occur in the chronic phase following TBI); neurology (to address issues related to mental status and cognition); PM&R (to assess vision and balance); and behavioral health (to assess cognitive and psychosocial needs). Other providers familiar with TBI may be incorporated into the care team as necessary based on the symptoms of an individual patient. Referrals do not need to stem from the providers caring for the patient during initial presentation. Rather, any provider caring for a patient with TBI should be aware of the complexity of possible symptoms and should refer to appropriate specialties as needed.

TBI, traumatic brain injury; GHD, growth hormone deficiency; ICU, intensive care unit; PCP, primary care physician; PM&R, physical medicine and rehabilitation.

### Assessment and treatment of GHD in the transition phase

The transition phase is a broadly defined period typically beginning in late puberty and ending in adulthood, approximately 6 to 7 years after the achievement of adult height.^26,77^ This phase is marked by substantial physical and psychosocial changes including cessation of linear growth, sexual maturation, and cognitive development, and a push for independence. These factors, along with the requirement to manage chronic illnesses and migrate management from pediatric to adult healthcare systems, combine to make the transition period a high-risk time during which many individuals may discontinue treatment and/or be lost to follow-up care.^116^ Poorly managed transitions have been shown to increase morbidity and mortality^76,116^ and three national surveys conducted in the U.S. revealed that 60% of patients with specialized healthcare needs and their families do not receive the required support for a successful transition to adult healthcare services.^116^

As endogenous production of GH and IGF-I declines during the transition phase, GH status should be reevaluated at this time.^77^ Further, pituitary deficiencies post-TBI can develop at a delay: evaluation of pituitary status, especially GH production, may be prompted by new symptoms during the transition phase. Current recommendations suggest retesting patients in the transition phase for GHD using GH stimulation tests when there is clinical suspicion of GHD and when serum IGF-I SDS is <0.^76,117^ Because most transition-age patients with GHD are asymptomatic, the authors recommend that patients with serum IGF-I SDS ≥0 be followed for evidence of symptoms or body composition changes. Testing is indicated after completion of longitudinal growth and at least 1 month after discontinuation of GH therapy.^76,77,102,117^ Children who have completed linear growth early may need to be retested for GHD using the pediatric testing options described above. In general, the number of stimulation tests required to confirm GHD in transition-age individuals is dependent on the level of suspicion for GHD.^76^ Retesting is not required in patients with ≥3 pituitary hormone deficiencies and with low serum IGF-I (< -2.0 SDS), as GHD is likely in these patients.^73,76,102^ As in initial pituitary hormone analyses, GH stimulation tests should be performed after diagnoses and treatments for all other pituitary hormone deficiencies have been established.^76,77^

Specific tests and cut points for confirming GHD in transition-age patients have not been extensively studied.^102^ The ITT (GHD threshold ≤5 μg/L),^76,77,88^ GST (GHD thresholds <3 μg/L for normal-BMI patients, and overweight patients with BMIs between 25-30 kg/m^2^ and a high pretest probability of GHD and <1 μg/L for obese patients with BMI >30 kg/m^2^ and overweight patients with BMIs between 25-30 kg/m^2^ and a low pretest probability of GHD),^76^ and macimorelin tests (GHD threshold ≤2.8 μg/L, only approved for patients aged ≥18 years)^76^ are recommended for diagnosis of GHD in adult patients. However, the ITT is used less frequently due to its contraindications and safety concerns.^76^ Arginine, clonidine, and l-DOPA have been shown to have low sensitivity in transition-age and adult patients and are therefore not recommended for confirmatory testing during transition.^76,118^

The predictive value of serum IGF-I concentrations alone to detect GHD in the transition phase has proven to be disappointing. One report showed that using the common threshold of IGF-I ≤ -2 SDS to indicate GHD would have excluded over one-third of transition-age study patients found to have GHD.^119^ Instead, IGF-I concentration measurements should be used only in conjunction with stimulation tests to determine GHD. Biochemical assay results can also be affected by nutritional and pubertal status; there is no consensus on priming with sex steroids prior to testing for GHD in transition-age patients.^75^ Although GH provocation tests are considered the mainstay of GHD diagnosis in transition-age patients, issues with variability, reproducibility, and limited evidence for cutoff values in these patients mean that results should be interpreted cautiously.^75^

Besides holding for retesting purposes, it is recommended to continue GH therapy in patients with confirmed GHD during the transition period, even if adult height has been achieved, as treatment is associated with improved body composition, bone health, cognitive function, QOL, and lipid metabolism in adulthood.^73,76,92,98,120^ Patients who remain GH deficient after reaching adult height can be at risk of adverse metabolic outcomes, increased fat mass and BMI, decreased bone mineralization and mineral density, and decreased QOL if GH therapy is stopped.^73,76,82,121-123^ The optimal length of time for GH treatment in transition-phase individuals and adults is unclear. In general, if the patient continues to see clinical benefit from the treatment, it may have to be continued throughout adulthood.^76^ In some situations, the patient's partner, caregiver, or family member may be an important historian of the continued symptomatic benefits of GH treatment, as individuals with TBI and some otherwise healthy adults with GHD may not recognize these treatment benefits themselves.

During the transition phase, current guideline recommendations support resuming GH dosing at approximately 50% of the last-used pediatric dose, with adjustments made thereafter to keep IGF-I between 0 and +2 SDS.^76,102^ Some individuals may require higher GH doses during puberty and young adulthood; however, these doses typically decrease with age and as IGF-I concentrations naturally fall.^76^ IGF-I should be monitored at least every 6 months^77^ and the GH dose should be adjusted based on serum IGF-I, clinical response, and any side effects (e.g., headache, hyperglycemia) that may occur.^76,102^ Current guidelines suggest that patients be monitored annually for blood pressure, body measurements (height, weight, BMI, hip and waist circumference), lipid profiles, and bone mineral density.^76^ Serum glucose, hemoglobin A1c, and laboratory parameters related to other pituitary hormone deficits should also be monitored.^102,117^ In their own clinical practice, the authors of this review note that they obtain annual readings of most of the parameters mentioned above but may also obtain additional readings, including liver enzymes, comprehensive metabolic panels, and thyroid function tests. Choice of specific tests ordered may vary based on factors including the final adult height of the patient, length of time since TBI, age, and medical history. A few authors note that they also obtain body composition (in late-stage adolescents) and bone mineral density by DEXA (dual-energy x-ray absorptiometry); however, this can be complicated by cost and availability.

### Transition phase challenges

There are many challenges to transitioning a pediatric patient with a history of TBI and GHD to adult services. One barrier to adequate care and appropriate referrals to adult endocrinologists is the limited awareness (among both patients and providers) of the need to continue GH treatment in transition-age and adult patients.^76,123^ Given the misconception that GH is important only for attaining adult height, there is a risk that a patient may discontinue GH treatment while under pediatric care or may stop seeing a pediatric endocrinologist altogether without realizing the importance of adult GH replacement and endocrinology care.^82,123^ In the experience of these authors, patients with GHD often stop GH treatment for a period of time during the transition phase; however, there are insufficient data to report on the number of patients who are retested and who eventually resume GH treatment. Emerging data continue to show that GH treatment can improve non–height-related parameters such as bone mass, body composition, and QOL, even after adult height has been reached. Therefore, it is crucial that patients with TBI and GHD visit an adult endocrinologist to evaluate and treat GHD in the transition phase.^70,76,77,82,117,122^

A lack of communication and coordination between pediatric and adult healthcare providers can result in inefficient treatment, patients being lost to follow-up as adults, or a large gap between the end of pediatric care and the beginning of adult care.^117,123-125^ In one study, the median time between the end of GH treatment in pediatric care and first evaluation in adult care was 3.4 years^120^; as discussed above, this gap in treatment can have negative consequences on metabolism, body composition, and bone density.^82^

Insurance coverage issues can also severely limit a patient's access to appropriate transitional and adult healthcare.^116,123,125^ The transition phase occurs at a time when many young adults will age out of their parents' health insurance plans, and it is therefore critical for these individuals to obtain adequate coverage. Although only 3.5% of young adults with special healthcare needs were uninsured in 2010, 34.3% did not have adequate coverage to meet their needs.^126^

In general, the more complex a patient's condition is, the greater the negative impact that condition has on the ease of transition.^127^ The transition phase can be made more complicated by unrecognized and insufficiently treated concomitant deficiencies in pituitary hormones besides GH, as affected patients will need to optimize and continue multiple hormone treatments during the switch from pediatric to adult care. Additionally, patients with cognitive difficulties (such as may stem from the TBI) may be incapable of managing their own care and may require assistance from transition care coordinators, caregivers, and/or family members.

To achieve appropriate continuity of care during the transition phase, it is important that providers begin counseling patients and caregivers on transition strategies as early as possible. Ideally, this should be at approximately 11 to 12 years of age or at the time of diagnosis. Generalized programs such as the Ready Steady Go program in the U.K. and the Got Transition initiative in the U.S. have been developed to assess patient readiness and indicate knowledge gaps prior to transition from pediatric to adult care^128,129^ and may also help to facilitate smooth transition for patients with TBI and GHD. Endocrinology-specific tools have also been developed, including the Endocrine Society readiness questionnaire and the Pediatric Endocrine Society transition toolkit.^123^ It can be helpful to develop a documented transition plan that details all the steps that need to be taken during the move to adult services.^76,127,129^ In addition, it has been suggested that adapting modes of communication to those preferred by younger patients (e.g., phone apps and text messaging) may contribute to improved transitions to adult healthcare offices.^123^

It is important that pediatric providers communicate directly with adult providers to facilitate the seamless transition of their patients.^76^ Direct communication can involve conversations between physicians, transfer of health records, and referrals to adult providers.^123^ Formal meetings or joint healthcare visits attended by both pediatric and adult providers can also improve continuity during the transition of care.^123^ As has been shown in other chronic conditions, including diabetes mellitus and cystic fibrosis, dedicated transition clinics staffed by pediatric and adult providers and transition care coordinators can increase communication and make the process more seamless. Unfortunately, these clinics may not be available to all patients for socioeconomic and/or geographic reasons.^76,77,125^ Regardless of the availability of transition care clinics and coordinators, patients with TBI and GHD should establish care with one provider (typically their PCP or endocrinologist) to act as a single point of contact during their transition to adult services. Notably, a 2019 advisory board suggested that the pediatric endocrinologist should play a central role in the transition of patients with GHD without TBI, including providing access to educational materials and developing a specific plan for the patient's transfer to adult care.^123^ It seems likely that the pediatric endocrinologist could play a similarly effective role in the transition of care for patients with both TBI and GHD.

### Conclusion

The occurrence of post-TBI GHD in pediatric, adolescent, and transition-age patients is underappreciated by many healthcare providers and its diagnosis and management has been incompletely studied to date. While multiple GHD diagnostic tests are available to children and young adults, there is a lack of consensus as to sensitive and specific thresholds for indicating GHD in this population. Similarly, well-defined diagnostic thresholds are lacking in the transition phase. Retesting for GHD is critical during this period, as it will inform the possible resumption of GH treatment and subsequent effects on body composition, metabolic status, and QOL. The transition phase is a complex period limited by few formal guidelines, minimal communication between providers, substandard counseling of patients and families, and inadequate insurance coverage. With this review, we aim to increase understanding of the diagnostic and treatment challenges facing patients with TBI and GHD during the transition phase, focus attention on the need for GH therapy beyond attaining adult height, and encourage providers to maintain awareness of the complex, multi-disciplinary needs of these patients.

## Supplementary Material

Supplemental data

Supplemental data
