# Supplementary material for: Growth Hormone Deficiency Following Traumatic Brain Injury in Pediatric and Adolescent Patients: Presentation, Treatment, and Challenges of Transitioning from Pediatric to Adult Services
Source: J Neurotrauma. 2023 Jun 27;40(13-14):1274–85. doi: 10.1089/neu.2022.0384 (PMC10294565; doi:10.1089/neu.2022.0384)
Supplement: Supplemental data [file Suppl_TableS1.docx]

**Supplemental Table 1. Prevalence of PTHP in the chronic phase after TBI in children and adolescents.** Studies included in this table were those that reported only anterior pituitary deficiencies in the chronic phase after TBI. Studies that reported non-anterior pituitary deficiencies, including diabetes insipidus, were excluded. If possible, for studies that reported both anterior and non-anterior pituitary deficiencies, prevalence rates for anterior pituitary deficiencies were extracted and reported here. GHD was required to be diagnosed using a stimulation test, adhering to the cutoff value of <10 μg/L suggested in the GH Research Society guidelines published in 2000.^1^ For studies that reported GHD prevalence rates based on more or less stringent thresholds, these guidelines were retroactively applied and the resulting prevalence rates reported in this table. *ACTH, adrenocorticotropic hormone; FSH, follicle-stimulating hormone; FT_4_, free [unbound] thyroxine; GCS, Glasgow Coma Score; GH, growth hormone; GHD, growth hormone deficiency; LH, luteinizing hormone; PTHP, post-traumatic hypopituitarism; TBI, traumatic brain injury; TSH, thyroid-stimulating hormone.*

| **Citation** | **N** | **Deficiencies reported** | **% PTHP** | **Notes** |
| --- | --- | --- | --- | --- |
| Aimaretti G, et al. (2005)^2^ | 23 | GHD, secondary hypogonadism, secondary hypocortisolism | 3 months: 30%  12 months: 30% | *TBI severity (based on GCS):*  Mild: 10 patients  Moderate: 6 patients  Severe: 7 patients |
| Bellone S, et al. (2013)^3^ | 70 | GHD, FSH/LH deficiency, secondary hypocortisolism, secondary hypothyroidism, TSH deficiency, precocious puberty | 10% | *TBI severity (based on GCS):*  Mild: 40 patients  Moderate: 11 patients  Severe: 19 patients |
| Casano-Sancho P, et al. (2013)^4^ | 37 | GHD | 3 months: 47.8%  12 months: 34.7% | *TBI severity (based on GCS):*  Mild: 8 patients  Moderate: 7 patients  Severe: 22 patients |
| Daskas N, et al. (2019)^5^ | 25 | GHD, suboptimal cortisol response | 28% | - |
| Heather NL, et al. (2012)^6^ | 198 | GHD, ACTH deficiency, precocious puberty, hypothyroidism | 33% | *TBI severity (based on GCS):*  Mild: 109 patients  Moderate: 35 patients  Severe: 54 patients |
| Kaulfers AD, et al. (2010)^7^ | 6 months: 24  12 months: 21 | Low TSH surge, low FT_4_, precocious puberty | 6 months: 58%  12 months: 29% | - |
| Khadr SN, et al. (2010)^8^ | 33 | GHD, low cortisol, prolactin deficiency | 39% | *TBI severity (based on GCS):*  Mild: 6 patients  Moderate: 15 patients  Severe: 12 patients |
| Norwood KW, et al. (2010)^9^ | 32 | GHD | 31% | - |
| Personnier C, et al. (2014)^10^ | 87 | GHD, TSH deficiency, ACTH deficiency | 31% | Only included patients with severe TBI |
| Ulutabanca H, et al. (2014)^11^ | 22 | GHD, ACTH deficiency | 13.3% | - |

**References**

1. Growth Hormone Research Society. Consensus guidelines for the diagnosis and treatment of growth hormone (GH) deficiency in childhood and adolescence: Summary statement of the GH Research Society. J Clin Endocrinol Metab 2000;85(11),3990-3993. doi:10.1210/jcem.85.11.6984.

2. Aimaretti G, Ambrosio MR, Di Somma C, et al. Hypopituitarism induced by traumatic brain injury in the transition phase. J Endocrinol Invest 2005;28(11),984-989. doi:10.1007/BF03345336.

3. Bellone S, Einaudi S, Caputo M, et al. Measurement of height velocity is an useful marker for monitoring pituitary function in patients who had traumatic brain injury. Pituitary 2013;16(4),499-506. doi:10.1007/s11102-012-0446-0.

4. Casano-Sancho P, Suarez L, Ibanez L, et al. Pituitary dysfunction after traumatic brain injury in children: Is there a need for ongoing endocrine assessment? Clin Endocrinol (Oxf) 2013;79(6),853-858. doi:10.1111/cen.12237.

5. Daskas N, Sharples P, Likeman M, et al. Growth hormone secretion, fatigue and quality of life after childhood traumatic brain injury. Eur J Endocrinol 2019;181(3),331-338. doi:10.1530/EJE-19-0166.

6. Heather NL, Jefferies C, Hofman PL, et al. Permanent hypopituitarism is rare after structural traumatic brain injury in early childhood. J Clin Endocrinol Metab 2012;97(2),599-604. doi:10.1210/jc.2011-2284.

7. Kaulfers AM, Backeljauw PF, Reifschneider K, et al. Endocrine dysfunction following traumatic brain injury in children. J Pediatr 2010;157(6),894-899. doi:10.1016/j.jpeds.2010.07.004.

8. Khadr SN, Crofton PM, Jones PA, et al. Evaluation of pituitary function after traumatic brain injury in childhood. Clin Endocrinol (Oxf) 2010;73(5),637-643. doi:10.1111/j.1365-2265.2010.03857.x.

9. Norwood KW, Deboer MD, Gurka MJ, et al. Traumatic brain injury in children and adolescents: Surveillance for pituitary dysfunction. Clin Pediatr (Phila) 2010;49(11),1044-1049. doi:10.1177/0009922810376234.

10. Personnier C, Crosnier H, Meyer P, et al. Prevalence of pituitary dysfunction after severe traumatic brain injury in children and adolescents: A large prospective study. J Clin Endocrinol Metab 2014;99(6),2052-2060. doi:10.1210/jc.2013-4129.

11. Ulutabanca H, Hatipoglu N, Tanriverdi F, et al. Prospective investigation of anterior pituitary function in the acute phase and 12 months after pediatric traumatic brain injury. Childs Nerv Syst 2014;30(6),1021-1028. doi:10.1007/s00381-013-2334-y.
