# Supplementary material for: Growth Hormone Deficiency Following Traumatic Brain Injury in Pediatric and Adolescent Patients: Presentation, Treatment, and Challenges of Transitioning from Pediatric to Adult Services
Source: J Neurotrauma. 2023 Jun 27;40(13-14):1274–85. doi: 10.1089/neu.2022.0384 (PMC10294565; doi:10.1089/neu.2022.0384)
Supplement: Supplemental data [file Suppl_TableS2.docx]

**Supplemental Table 2. Prevalence of GHD in the chronic phase after TBI in children and adolescents.** Studies included in this table reported rates of GHD in pediatric and adolescent patients in the chronic phase post TBI. Reports of GHD were limited to those that were diagnosed using a stimulation test and that adhered to the cutoff value of <10 μg/L suggested in the GH Research Society guidelines published in 2000.^1^ For studies that reported GHD prevalence rates based on more or less stringent thresholds, these guidelines were retroactively applied and the resulting prevalence rates reported in this table. *GCS, Glasgow Coma Score; GHD, growth hormone deficiency; GHRH, growth hormone releasing hormone; ITT, insulin tolerance test; l-DOPA, levodopa; TBI, traumatic brain injury.*

| **Citation** | **N** | **Stimulation test(s) used** | **Threshold applied** | **% GHD** | **Notes** |
| --- | --- | --- | --- | --- | --- |
| Aimaretti G, et al. (2005)^2^ | 23 | GHRH + arginine | <9.0 μg/L | 26% (3 months post TBI)  21.7% (12 months post TBI) | *TBI severity (based on GCS):*  Mild: 10 patients  Moderate: 6 patients  Severe: 7 patients |
| Casano-Sancho P, et al. (2013)^3^ | 23 | Glucagon, clonidine | <10 μg/L | 47.8% (3 months post TBI)  34.7% (12 months post TBI) | - |
| Daskas N, et al. (2019)^4^ | 25 | ITT | <3 μg/L (young adults)  <5 μg/L (transition age)  <6.7 μg/L (pediatric) | 24% | - |
| Heather NL, et al. (2012)^5^ | 198 | Arginine + clonidine | <10 μg/L | 33% | *TBI severity (based on GCS):*  Mild: 109 patients  Moderate: 35 patients  Severe: 54 patients |
| Khadr SN, et al. (2010)^6^ | 33 | ITT, glucagon | <5 μg/L | 21% | *TBI severity (based on GCS):*  Mild: 6 patients  Moderate: 15 patients  Severe: 12 patients |
| Niederland T, et al. (2007)^7^ | 26 | l-DOPA, ITT | <7 μg/L | 42% | - |
| Norwood KW, et al. (2010)^8^ | 32 | Arginine + glucagon | <5 μg/L (≥18 years)  <7 μg/L (<18 years) | 31% | - |
| Personnier C, et al. (2014)^9^ | 87 | Glucagon, betaxolol + glucagon, arginine, arginine + insulin | <7 μg/L (required 2 tests) | 31% | Only included patients with severe TBI |
| Ulutabanca H, et al. (2014)^10^ | 22 | GHRH + arginine | <7 μg/L | 9.1% | - |

**References**

1. Growth Hormone Research Society. Consensus guidelines for the diagnosis and treatment of growth hormone (GH) deficiency in childhood and adolescence: Summary statement of the GH Research Society. J Clin Endocrinol Metab 2000;85(11),3990-3993. doi:10.1210/jcem.85.11.6984.

2. Aimaretti G, Ambrosio MR, Di Somma C, et al. Hypopituitarism induced by traumatic brain injury in the transition phase. J Endocrinol Invest 2005;28(11),984-989. doi:10.1007/BF03345336.

3. Casano-Sancho P, Suarez L, Ibanez L, et al. Pituitary dysfunction after traumatic brain injury in children: Is there a need for ongoing endocrine assessment? Clin Endocrinol (Oxf) 2013;79(6),853-858. doi:10.1111/cen.12237.

4. Daskas N, Sharples P, Likeman M, et al. Growth hormone secretion, fatigue and quality of life after childhood traumatic brain injury. Eur J Endocrinol 2019;181(3),331-338. doi:10.1530/EJE-19-0166.

5. Heather NL, Jefferies C, Hofman PL, et al. Permanent hypopituitarism is rare after structural traumatic brain injury in early childhood. J Clin Endocrinol Metab 2012;97(2),599-604. doi:10.1210/jc.2011-2284.

6. Khadr SN, Crofton PM, Jones PA, et al. Evaluation of pituitary function after traumatic brain injury in childhood. Clin Endocrinol (Oxf) 2010;73(5),637-643. doi:10.1111/j.1365-2265.2010.03857.x.

7. Niederland T, Makovi H, Gal V, et al. Abnormalities of pituitary function after traumatic brain injury in children. J Neurotrauma 2007;24(1),119-127. doi:10.1089/neu.2005.369ER.

8. Norwood KW, Deboer MD, Gurka MJ, et al. Traumatic brain injury in children and adolescents: Surveillance for pituitary dysfunction. Clin Pediatr (Phila) 2010;49(11),1044-1049. doi:10.1177/0009922810376234.

9. Personnier C, Crosnier H, Meyer P, et al. Prevalence of pituitary dysfunction after severe traumatic brain injury in children and adolescents: A large prospective study. J Clin Endocrinol Metab 2014;99(6),2052-2060. doi:10.1210/jc.2013-4129.

10. Ulutabanca H, Hatipoglu N, Tanriverdi F, et al. Prospective investigation of anterior pituitary function in the acute phase and 12 months after pediatric traumatic brain injury. Childs Nerv Syst 2014;30(6),1021-1028. doi:10.1007/s00381-013-2334-y.
